# Characteristics of Patients Not Receiving Chemical Thromboprophylaxis Following Foot and Ankle Surgery: Data From the Multicenter, Prospective UK Foot and Ankle Thrombo-Embolism Audit (UK-FATE)

**DOI:** 10.1177/10711007241258159

**Published:** 2024-06-13

**Authors:** Karan Malhotra, Linzy Houchen-Wolloff, Lyndon Mason, Jitendra Mangwani, UK-FATE Collaborative

**Affiliations:** 1 Foot & Ankle Unit, Royal National Orthopaedic Hospital, Stanmore, United Kingdom; 2 Department of Orthopaedics & Musculoskeletal Science, University College London, London, United Kingdom; 3 University Hospitals of Leicester NHS Trust, Leicester, United Kingdom; 4 Liverpool University Hospitals NHS Foundation Trust, Liverpool, United Kingdom; 5 University of Liverpool, Liverpool, United Kingdom; 6 Academic Team of Musculoskeletal Surgery, University Hospitals of Leicester, Leicester, United Kingdom

**Keywords:** venous thromboembolism, thromboprophylaxis, foot; ankle, surgery, UK-FATE

## Abstract

**Background:**

Although the rate of venous thromboembolism (VTE) after foot and ankle surgery is low, multiple factors influence risk for individual patients. Furthermore, there are no clear guidelines on which patients may benefit from chemical thromboprophylaxis. Our aim was to assess patients not treated with chemical thromboprophylaxis after foot and ankle surgery, and to report on their specific patient and surgical risk factors for VTE.

**Methods:**

This was a multicenter, prospective, national audit of patients undergoing foot and ankle surgery (including Achilles tendon ruptures) from 68 participating UK centers. The study was conducted between June 1, 2022, and November 30, 2022, with a further 3-month follow-up. Following data cleansing, 3309 patients were included who did not receive postoperative thromboprophylaxis.

**Results:**

Most patients were elective cases (2589 patients, 78.24%) with ASA grade I or II (2679 patients, 80.96%), fully weightbearing postoperatively (2752 patients, 83.17%), and either without ankle splintage, or splinted in a plantigrade boot (2797 patients, 84.53%). The VTE rate was 0.30% overall (11 cases), with no VTE-related mortality. No single demographic, surgical, or postoperative factor was associated with reduced risk of VTE. However, patients who had elective or trauma surgery not involving the ankle, who were ASA grade I or II and who were weightbearing immediately postoperatively (without splinting or in a plantigrade boot) had a VTE rate of 0.05% (1 of 1819 patients), compared with 0.67% (10 of 1490 patients, *P* = .002).

**Conclusion:**

Patients not receiving chemical thromboprophylaxis had a low incidence of symptomatic VTE, although they do represent a curated group considered lower risk. Within this group we describe characteristics associated with a substantially lower risk of VTE. All patients should be assessed on an individual basis, and further work is required to substantiate our findings.

## Graphical abstract

**Graphical Abstract:**
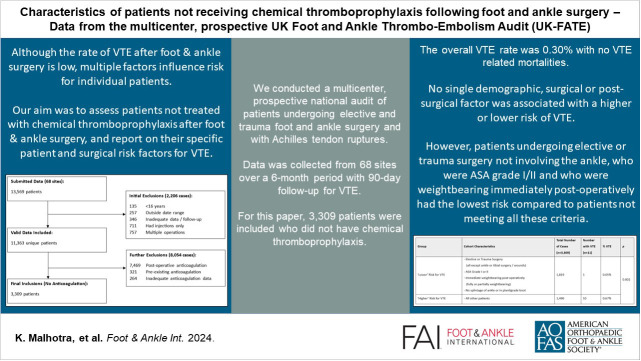
This is a visual representation of the abstract.

**Level of Evidence:** Level II, prospective cohort study.

## Introduction

Symptomatic venous thromboembolism (VTE) is a potentially devastating complication of foot and ankle trauma and surgery. Although the risk is generally accepted as low,^2,6,7^ numerous studies have aimed to quantify the incidence and risk factors for VTE,^1,10,12^ and strived to determine whether chemical thromboprophylaxis can mitigate these risks.^2-4,14,15^ Although consensus is lacking on many aspects surrounding VTE, elective foot and ankle surgery and forefoot trauma have been shown to have a lower risk of VTE.^3,6,7,12^ Conversely, several studies demonstrate that Achilles tendon ruptures are associated with a higher risk of VTE than other groups of patients.^1,2,7,10^

Testroote et al^14^ report that chemical thromboprophylaxis can reduce the risk of VTE in patients immobilized in a lower limb cast, although it is not clear whether this translates to patients who can bear weight and other authors have conversely found that prophylaxis makes no difference to VTE rate in immobilized patients.^15^ In the United Kingdom, the National Institute for Health and Care Excellence (NICE) published guidance (NG89) on the thromboprophylaxis of patients with lower limb immobilization and following foot and ankle surgery.^8^ Their recommendations are to consider the use of chemical thromboprophylaxis in these patient groups if being immobilized or having surgery that lasts longer than 90 minutes.

With a wealth of often conflicting literature available, there remains little consensus to guide surgeons as to whether an individual patient will benefit from chemical thromboprophylaxis. This may mean that surgeons choose to err on the side of caution and prescribe chemical anticoagulants that may not be required. To help obtain consensus, it may be useful to examine the characteristics of a diverse cohort of patients who were assessed in a “real-world” setting as not requiring chemical thromboprophylaxis.

The primary aim of this study was therefore to analyze and report on characteristics of a cohort of patients undergoing foot and ankle surgery who did not receive chemical thromboprophylaxis postoperatively. Secondary aims included analyzing specific demographic, operative, and management characteristics that may influence the risk of symptomatic VTE.

## Methods

This paper is a subanalysis of data obtained from the United Kingdom Foot and Ankle Thromboembolic Audit (UK-FATE Audit). The UK-FATE was a prospective, multicenter, national audit carried out across the United Kingdom. Data were collected on all patients having a foot and ankle procedure in an operating theatre from participating centers during the study period. Patients were also included if they had a nonoperatively managed Achilles tendon rupture. The data collection began on June 1, 2022, and ended on November 30, 2022, with a further 3-month period for follow-up. Each site registered the project as a local audit with appropriate approvals and the project was also registered as an audit at the lead center (Leicester University Hospitals, Ref: 11908a).

### Data Collection

Each site was sent a standardized, encrypted spreadsheet for data collection. Data were collected on all patients, whether they were treated with chemical thromboprophylaxis or not. Data collected included demographic data, type of surgery (elective, trauma, acute diabetic foot, and Achilles tendon rupture), urgency of surgery, ASA grade, comorbidities, thromboprophylaxis (chemical and mechanical), operative data, postoperative rehabilitation and splinting, complications, symptomatic 90-day VTE rate (radiologically confirmed), and mortality. The modality to be used for radiologic diagnosis was not stipulated, but sites were encouraged to identify whether a VTE had been diagnosed through the following sources: review of notes and imaging at the local hospital, analysis of national hospital statistics data (to identify patients with VTE diagnosed at different centers), and by calling the patients at 90 days after surgery (or after Achilles tendon rupture) and asking them directly whether they had been diagnosed with a VTE. All data including comorbidities was reported as an absence or presence of the variable according to a provided datasheet key. A summary of all data columns and the keys are provided in the Supplementary Material (Appendix 1). Completed data were sent securely to the lead center, where it was uploaded to the Research Electronic Data Capture application (REDCap, Vanderbilt, TN).

### Patient Selection

**Figure 1.**
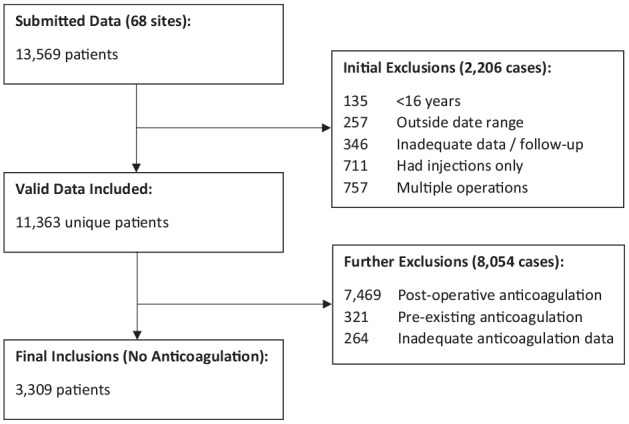
PRISMA flowchart for inclusion and exclusion criteria.

A total of 13 569 cases were submitted from 68 sites; after applying our initial exclusion criteria (Figure 1), 11 363 patients were included for subsequent analysis. Of these, 3630 did not receive any new postoperative anticoagulation and these cases were further examined for inclusion in this study. The decision to omit chemical prophylaxis was made by the treating unit in accordance with their local VTE risk assessment policies. Within this group, 321 patients were on preoperative anticoagulation (aspirin or clopidogrel) which was continued postoperatively and were excluded. A further 264 patients had inadequate anticoagulation data, leaving a total of 3309 patients available for final analysis (29.12%; Figure 1).

### Data Reporting and Statistical Analysis

Data were analyzed using IBM SPSS Statistics for Windows, v26 (IBM Corp, Armonk, NY). Data are presented as a descriptive analysis with percentages. Means are presented along with standard deviations. To establish whether there were significant differences in VTE rates between groups with categorical data, a Pearson χ^2^ test was used. To establish whether there were any factors that particularly influenced VTE rate in this group, multinomial logistic regression was used with the following factors included in the model: category of procedure (elective, trauma, diabetic, and Achilles tendon rupture), ASA grade, number of comorbidities (none, 1, more than 1), type of surgery (open or percutaneous), CEPOD urgency, use of tourniquet, use of mechanical prophylaxis, postoperative weightbearing status, postoperative splintage, patient age, and length of surgery. Using the factors identified as significantly affecting VTE risk, patients were divided into “higher” and “lower” risk groups for VTE post hoc, and the VTE rate was compared using a χ^2^ test. A *P* value of <.05 was considered significant.

## Results

Of the 3309 patients included, 1366 were male (41.28%) and the mean age was 60.0 ± 17.66 (range, 16-94 years) years. The breakdown of types of cases is shown in Table 1. Most patients in this group were elective (2589 patients, 78.24%) with ASA grade I or II (2679 patients, 80.96%), 1 or fewer comorbidities (2810 patients, 84.92%), fully weightbearing postoperatively (2752 patients, 83.17%), and without ankle splintage, or splinted in a plantigrade boot (2797 patients, 84.53%). Key demographic features are summarized in Table 2. The average length of surgery was 56.0 ± 39.62 minutes.

**Table 1.** Breakdown of Type of Cases in Patients Not Receiving Anticoagulation and the VTE Rates per Group.

Type of Case	Total Number of Cases (n = 3309)	Number With VTE (n = 11)	% VTE
Elective cases	2589	4	0.15
Elective (forefoot)	1689	2	0.12
Elective (ankle)	509	2	0.39
Elective (other)	391	–	–
Achilles tendon ruptures	133	4	3.01
Achilles tendon rupture (nonoperative)	128	4	3.12
Achilles tendon rupture (operative)	5	–	–
Trauma cases	508	3	0.59
Trauma (ankle or pilon)	192	2	1.04
Trauma (wound debridement)	114	1	0.88
Trauma (other)	202	–	–
Acute diabetic foot cases	79	**–**	**–**
Diabetic feet (all procedures)	79	–	–

Abbreviation: VTE, venous thromboembolism.

**Table 2.** Key Demographic and Treatment Factors Observed in This Patient Group.

Demographic	Number (n = 3309)	%
Gender		
Male	1366	41.28
Female	1943	58.72
Type of procedure		
Open surgery	2825	85.37
Percutaneous / Ex-Fix	355	10.73
N/A (Achilles rupture, nonoperative)	128	3.87
Urgency		
Elective	2555	77.21
Expedited	283	8.55
Urgent	339	10.24
Immediate	4	0.12
N/A (Achilles rupture, nonoperative)	128	3.87
Tourniquet		
Used	2467	74.55
None	842	25.45
Anaesthesia type		
General	2537	76.67
Regional	348	10.52
Local	287	8.67
None	137	4.14
ASA grade		
I	994	30.04
II	1685	50.92
III/IV/V	341	10.31
N/A or not specified	289	8.73
Significant comorbidities		
None	2055	62.10
1	755	22.82
2 or more	409	12.36
Not specified	90	2.72
Postoperative weightbearing status		
Fully weightbearing	2752	83.17
Partially weightbearing	267	8.07
Nonweightbearing (any duration)	294	8.88
Postoperative splintage (ankle)		
None	2459	74.31
Boot (plantigrade)	338	10.21
Boot (equinus)	224	6.77
Cast (plantigrade)	218	6.59
Cast (equinus)	32	0.97

Abbreviations: ASA, American Society of Anesthesiologists; N/A, not available.

### Incidence of Venous Thromboembolism

VTE occurred in 11 patients in this group (0.30%): 5 cases were of deep vein thrombosis (DVT) in the distal operated limb, 3 cases were of DVT in the proximal operated limb, 2 were cases of pulmonary embolism (PE; 1 bilateral), and 1 was a case of combined PE and DVT. Four of these VTE events were in Achilles tendon ruptures managed nonoperatively, 3 were in trauma cases, and 4 were in elective cases. Further details are given in Table 1. Nine patients with VTE were male, and the mean age of these patients was 51.8 (range, 34-71) years. Where VTE occurred, the mean time to VTE was 20.0 (range, 5-55) days.

χ^2^ analysis demonstrated a significant difference between VTE rates by type of diagnosis (elective, trauma, Achilles ruptures, acute diabetic feet, *P* < .001), but no significant differences between type of elective procedure, type of surgery (open/percutaneous), splintage, weightbearing status, ASA grade, or comorbidities. Regression analysis did not identify any single factor associated with an increased risk of VTE in this group of patients (Appendix 2).

### Identifying a Lower-Risk Group

As no single factor predicted VTE independently, we proceeded to identify a combination of factors that appeared to be associated with a lower VTE rate. To do this, we examined the cross-tabulation results between VTE and examined factors, and identified which factors had a higher or lower rate of VTE, even if not statistically significant. The factors thus noted to have the lowest rate of VTE were collated to create a “lower risk” group, which we then proceeded to examine post hoc. This included patients with elective and trauma surgery that did not involve the ankle, patients who had a lower ASA grade (I or II), and patients who were immediately weightbearing postoperatively (either in a splint or in a plantigrade boot). A total of 1819 patients (54.97%) met these criteria, and among them there was only 1 case of VTE (0.05%). This contrasted significantly from the rest of the cohort where the VTE rate was 0.67% (*P* = .002). Our selection criteria for this analysis are summarized in Table 3.

**Table 3.** Comparison of VTE Rate Between a Potentially Lower Risk Group vs the Rest of the Cohort, When Treated Without Chemical Thromboprophylaxis.^a^

Group	Cohort Characteristics	Total Number of Cases (n = 3309)	Number with VTE (n = 11)	% VTE	*P*
“Lower” risk for VTE	Elective or trauma surgery (all except ankle or tibial surgery/wounds) ASA grade I or II Immediate weightbearing postoperatively (fully or partially weightbearing) No splintage of ankle or in plantigrade boot	1819	1	0.05	.002
“Higher” risk for VTE	All other patients	1490	10	0.67	

Abbreviations: ASA, American Society of Anesthesiologists; VTE, venous thromboembolism.

a Patients were divided into 2 groups based on the factors listed and compared using a χ^2^ test.

### Mortality Rate

There were 5 cases of postoperative deaths within 90 days (0.15%). There were no cases of VTE-related mortality.

## Discussion

Our primary aim was to present a descriptive analysis of the patients from the UK-FATE study who were managed without chemical thromboprophylaxis. Overall, in this group the VTE rate was low at 0.30%, with no VTE-related mortality. In the current climate where the use of VTE prophylaxis is common practice, this group likely represents a curated cohort who were deemed by the treating clinicians as “low risk” for VTE. This may explain why there were no clear differences between various treatment modalities, patient factors, and VTE rate. The only difference appeared to be between elective patients and other cases where elective patients had a VTE rate of 0.15%, which was significantly lower than the rest. However, most of these patients were forefoot cases who did not have splintage postoperatively and who were fully weightbearing immediately postoperatively.

As part of our secondary aim, we further analyzed these relationships with regression analysis. Category of procedure did not appear to directly influence the rate of VTE, suggesting that other factors (such as weightbearing status, splintage, etc) may play a more important role than type of surgery. That the VTE rate is low overall suggests that the decision to omit chemical prophylaxis was appropriate in most cases. However, it is not possible to draw firm conclusions as to which patients are at the lowest risk of VTE. We are able, however, to identify the patient and operative characteristics most seen in this cohort, and which we have reported. Cases with the lowest rate of VTE were non-ankle elective and trauma cases with ASA grade I or II, immediately weightbearing postoperatively (fully or partially), and either without postoperative splintage, or in a plantigrade boot. These patients represented more than half the reported patients and had a VTE rate of 0.05%. This would suggest that this cohort of patients may be safely managed without chemical VTE prophylaxis postoperatively although further work is required to confirm this. Managing these patients without chemical thromboprophylaxis could help avoid the risks associated with chemical prophylaxis and provide a cost saving.

Our findings are broadly supported by previous literature. Jameson et al^6^ performed a retrospective analysis of VTE events in the United Kingdom and found a VTE rate of 0.03% for first ray surgery and 0.14% for hindfoot surgery. Although their rates of reported VTE are lower than ours, their figures are likely an underestimate as it was based on data harvested from clinical coding. They also did not have access to many specific risk factors such as weightbearing status and splintage. Conversely, Griffiths et al^3^ report a higher rate of VTE than we observe, but this may be due to the curated nature of patents in our study.

In contrast to other studies, in our study, certain characteristics do not appear to independently influence VTE risk, including use of tourniquet, open or percutaneous surgery, length of surgery, type of anesthesia, number of comorbidities, gender, and age. Jameson et al^6^ and Mangwani et al^7^ identified increasing age and multiple comorbidities as risk factors for VTE. Although we did not find age to be a risk factor, the observed association in other studies may in fact be linked to the increased comorbidities (and in turn ASA grade) seen in older patients. NICE has suggested that patients undergoing surgery of longer than 90 minutes’ duration should be considered for chemical thromboprophylaxis.^8^ Although we did not find an association between length of surgery and VTE rate, our mean duration of surgery was substantially lower at 56 minutes. This is likely due to the nature of the surgery, which would have included shorter operations, with less complex fractures/hindfoot work, and where patients are more likely to be fully weightbearing directly after their surgery. The recommendation for considering prophylaxis in those with longer surgery should therefore still be considered, but it is unclear whether it the duration of surgery or the nature of surgery in those cases that has the greater impact.

The rate of VTE was higher in Achilles tendon ruptures, trauma cases (particularly around the ankle and with wound debridement), and elective surgery around the ankle. This may be on account of immobilization/splintage, although this cannot be definitively proven from this data set. Mangwani et al^7^ found that immobilization and nonweightbearing status increased VTE risk. Similar findings have been noted by other authors.^2,9,10,14^ In their systematic review, Testroote et al^14^ do acknowledge that it is unclear whether the increased VTE rate seen in patients immobilized in a cast applies to patients who are truly weightbearing. Although there is some debate on whether chemical thromboprophylaxis can reduce the VTE rate in a number of clinical scenarios, some authors have reported that anticoagulation could halve the risk of VTE in immobilized patients.^5,11^ We did not find a higher risk of VTE in trauma around the forefoot and midfoot. Soohoo et al^13^ found a low rate of PE in patients with metatarsal fractures, although because they did not include DVT, the overall VTE rate may have been higher.

### Strengths and Limitations

The strength of this study is that we have many patients from multiple sites across the United Kingdom, which improves the generalizability of our findings as it provides a “real world” snapshot of practice in a diverse cohort of patients. We were also able to identify a clear subgroup of patients who appeared to have a lower risk of VTE when managed without chemical thromboprophylaxis. However, this study has several limitations. Although it was a prospective study, there was no specific protocol that has been followed for deciding which patients receive prophylaxis. Therefore, the group in question has already been considered as low risk by the treating surgeons and so any statistical analysis will be skewed. We do not know which specific factors influenced the choice to omit anticoagulation, but all sites reported using a national or local VTE assessment tool in their decision making. It is also not clear how many other patients who met our criteria for lower risk would have been given prophylaxis, and how many patients who were given prophylaxis may not have needed it. We have only reported the incidence of symptomatic VTE, and there may be a much larger cohort of patients with asymptomatic VTE, which this study does not address. However, as these patients were followed up for 90 days, if the VTE remained asymptomatic for that duration, it may be less likely to be of clinical significance. Although we have tried to identify a subgroup of patients at lower risk of VTE, this needs to be interpreted with caution as this is a purely observational study, and therefore not explicitly set up to delineate this information. A randomized controlled study controlling for these factors is required to confirm our findings, and our work can serve as a basis for planning such a project. Finally, the numbers of VTE are very low and this limits the reliability of more in-depth analyses. Furthermore, the rarity of observed VTE may be another possible reason for the lack of observed association between VTE and individual characteristics, and this should be borne in mind when considering our results.

## Conclusion

We present a cohort of patients who underwent foot and ankle surgery and who were managed without chemical thromboprophylaxis postoperatively. Within this cohort, patients undergoing elective and trauma surgery not involving the ankle, who were ASA grade 1 or 2, and who were immediately weightbearing (either without splintage or in a plantigrade boot) had a symptomatic VTE rate of 0.05%. It is important to continue to assess each patient individually for VTE risk. These data may serve as the basis for further prospective comparative studies that are required to confirm our findings.

## Supplemental Material

sj-docx-2-fai-10.1177_10711007241258159 – Supplemental material for Characteristics of Patients Not Receiving Chemical Thromboprophylaxis Following Foot and Ankle Surgery: Data From the Multicenter, Prospective UK Foot and Ankle Thrombo-Embolism Audit (UK-FATE) Supplemental material, sj-docx-2-fai-10.1177_10711007241258159 for Characteristics of Patients Not Receiving Chemical Thromboprophylaxis Following Foot and Ankle Surgery: Data From the Multicenter, Prospective UK Foot and Ankle Thrombo-Embolism Audit (UK-FATE) by Karan Malhotra, Linzy Houchen-Wolloff, Lyndon Mason and Jitendra Mangwani in Foot & Ankle International

sj-docx-3-fai-10.1177_10711007241258159 – Supplemental material for Characteristics of Patients Not Receiving Chemical Thromboprophylaxis Following Foot and Ankle Surgery: Data From the Multicenter, Prospective UK Foot and Ankle Thrombo-Embolism Audit (UK-FATE) Supplemental material, sj-docx-3-fai-10.1177_10711007241258159 for Characteristics of Patients Not Receiving Chemical Thromboprophylaxis Following Foot and Ankle Surgery: Data From the Multicenter, Prospective UK Foot and Ankle Thrombo-Embolism Audit (UK-FATE) by Karan Malhotra, Linzy Houchen-Wolloff, Lyndon Mason and Jitendra Mangwani in Foot & Ankle International

sj-docx-4-fai-10.1177_10711007241258159 – Supplemental material for Characteristics of Patients Not Receiving Chemical Thromboprophylaxis Following Foot and Ankle Surgery: Data From the Multicenter, Prospective UK Foot and Ankle Thrombo-Embolism Audit (UK-FATE) Supplemental material, sj-docx-4-fai-10.1177_10711007241258159 for Characteristics of Patients Not Receiving Chemical Thromboprophylaxis Following Foot and Ankle Surgery: Data From the Multicenter, Prospective UK Foot and Ankle Thrombo-Embolism Audit (UK-FATE) by Karan Malhotra, Linzy Houchen-Wolloff, Lyndon Mason and Jitendra Mangwani in Foot & Ankle International

sj-pdf-1-fai-10.1177_10711007241258159 – Supplemental material for Characteristics of Patients Not Receiving Chemical Thromboprophylaxis Following Foot and Ankle Surgery: Data From the Multicenter, Prospective UK Foot and Ankle Thrombo-Embolism Audit (UK-FATE) Supplemental material, sj-pdf-1-fai-10.1177_10711007241258159 for Characteristics of Patients Not Receiving Chemical Thromboprophylaxis Following Foot and Ankle Surgery: Data From the Multicenter, Prospective UK Foot and Ankle Thrombo-Embolism Audit (UK-FATE) by Karan Malhotra, Linzy Houchen-Wolloff, Lyndon Mason and Jitendra Mangwani in Foot & Ankle International
