# Supplementary material for: Characteristics of Patients Not Receiving Chemical Thromboprophylaxis Following Foot and Ankle Surgery: Data From the Multicenter, Prospective UK Foot and Ankle Thrombo-Embolism Audit (UK-FATE)
Source: Foot Ankle Int. 2024 Jun 13;45(9):943–9. doi: 10.1177/10711007241258159 (PMC11408974; doi:10.1177/10711007241258159)
Supplement: sj-docx-2-fai-10.1177_10711007241258159 – Supplemental material for Characteristics of Patients Not Receiving Chemical Thromboprophylaxis Following Foot and Ankle Surgery: Data From the Multicenter, Prospective UK Foot and Ankle Thrombo-Embolism Audit (UK-FATE) [file sj-docx-2-fai-10.1177_10711007241258159.docx]

Reported Author Disclosures:

FAI-24-0008

Characteristics of patients not receiving chemical thromboprophylaxis following foot and ankle surgery - Data from the multicentre, prospective UK Foot and Ankle Thrombo-Embolism Audit (UK-FATE)

The authors report support for the present manuscript from Leicester Hospitals Charity Grant that was received for the amount of £4,930 (Ref: APP7681). This was to cover the costs of NIHR Biomedical Research Centre Data staff (at University Hospitals Leicester), the database platform, and statistician time. The payment was made directly to the institution and funders had no role in design, analysis or reporting of the project. Disclosure forms for all authors are available online.

Funding Information:

Leicester Hospitals Charity x

APP7681

Ethical Approval:

Ethical approval was not sought for the present study as it was conducted as a national Audit of practice. Each site individually applied for approval from their clinical governance department for collecting prospective data on routine clinical care. No intervention was carried out and therefore patient care was not affected.
