# Supplementary material for: Characteristics of Patients Not Receiving Chemical Thromboprophylaxis Following Foot and Ankle Surgery: Data From the Multicenter, Prospective UK Foot and Ankle Thrombo-Embolism Audit (UK-FATE)
Source: Foot Ankle Int. 2024 Jun 13;45(9):943–9. doi: 10.1177/10711007241258159 (PMC11408974; doi:10.1177/10711007241258159)
Supplement: sj-docx-3-fai-10.1177_10711007241258159 – Supplemental material for Characteristics of Patients Not Receiving Chemical Thromboprophylaxis Following Foot and Ankle Surgery: Data From the Multicenter, Prospective UK Foot and Ankle Thrombo-Embolism Audit (UK-FATE) [file sj-docx-3-fai-10.1177_10711007241258159.docx]

Characteristics of patients not receiving chemical thromboprophylaxis following foot and ankle surgery – Data from the multicenter, prospective UK Foot and Ankle Thrombo-Embolism Audit (UK-FATE)

**Appendices**

# **Appendix 1:** Variables Recorded on Datasheet

| Variable | Categories (Units) |
| --- | --- |
| Gender | Male  Female |
| Age | (Years) |
| Foot & Ankle Diagnosis | 01 - Trauma - Distal Tibia  02 - Trauma - Malleolar  03 - Trauma - Talus  04 - Trauma - Calcaneus  05 - Trauma - Cuboid  06 - Trauma - Cuneiforms  07 - Trauma - Metatarsals  08 - Trauma - Phalanges  09 - Trauma - Achilles tendon  10 - Trauma - Other foot and ankle tendon  11 - Trauma - Other foot and ankle procedure  12 - Trauma - Wound management  13 - Acute Diabetic Foot - Wound debridement  14 - Acute Diabetic Foot - Drainage  15 - Acute Diabetic Foot - Forefoot amputation  16 - Acute Diabetic Foot - Midfoot Amputation  17 - Acute Diabetic Foot - Hindfoot Amputation  18 - Acute Diabetic Foot - BKA or above  19 - Elective - Forefoot  20 - Elective - Midfoot  21 - Elective - Hindfoot  22 - Elective - Elective Tendon Procedure  23 - Elective - Ankle |
| Date of Injury (for Trauma) | (Date) |
| Date of Admission | (Date) |
| Date of Surgery | (Date) |
| Date of Discharge from Hospital | (Date) |
| Operation Type | 1 - MUA/plaster  2 - Percutaneous surgery  3 - External fixation  4 - Open surgery  5 – Injection |
| Length of Surgery | (Minutes) |
| Urgency of Surgery | 1 - Immediate  2 - Urgent  3 - Expedited  4 - Elective |
| Tourniquet Used | 0 - None  1 - Thigh  2 - Calf  3 - Ankle |
| Tourniquet Duration | (Minutes) |
| Anaesthesia Type | 1 - Local  2 - Regional  3 - General  4 - Combination (regional/general) |
| ASA Grade | 1  2  3  4  5 |
| Patient on pre-existing anticoagulation | 0 - None  1 - Aspirin  2 - Clopidogrel  3 - DOAC  4 - LMWH  5 - Fondaparinux  6 - Warfarin  7 - Other |
| Pre-Operative Mechanical Prophylaxis Type (Trauma) | 0 - None  1 - Compression Stockings  2 - Foot Pump  3 - Calf Pump  4 - Stockings + Pump |
| Pre-Operative Chemical Prophylaxis Duration (Trauma) | 0 - None  1 - Uninterrupted till surgery  2 - Till day before surgery  3 - Stopped greater than 1 day before surgery |
| Post-Operative Mechanical Thromboprophylaxis Type | 0 - None  1 - Compression Stockings  2 - Foot Pump  3 - Calf Pump  4 - Stockings + Pump |
| Perioperative Tranexamic Acid | 0 - No  1 - Yes |
| Intra-op bleeding event | 0 - No  1 - Yes |
| Mechanical Thrombo-prophylaxis of other side | 0 - No  1 - Yes |
| Duration of Mechanical Prophylaxis | 1 - Whilst admitted  2 - 1 week  3 - 2 weeks  4 - 2-4 weeks  5 - 4-6 weeks  6 - > 6 weeks |
| Chemical Thrombo-prophylaxis | 0 - None  1 - Aspirin  2 - Clopidogrel  3 - DOAC  4 - LMWH  5 - Fondaparinux  6 - Warfarin  7 - Other |
| Duration of Chemical Prophylaxis | (Weeks) |
| Weightbearing | 1 - FWB  2 - PWB  3 - NWB 0-2 weeks  4 - NWB 2-4 weeks  5 - NWB 4-6 weeks  6 - NWB > 6 weeks |
| Splintage | 0 - None  1 - Aircast Boot  2 - Vacoped / Boot with wedges  3 - Cast - Plantigrade  4 - Cast - Equinus |
| Bloods for monitoring PLT | 0 - No  1 - Yes |
| Post-Surgery / Achilles Rupture Thrombotic Event | 0 - None  1 - Symptomatic DVT - Operated / Injured Limb (Distal)  2 - Symptomatic DVT - Operated / Injured Limb (Proximal)  3 - Symptomatic DVT - Non-Operated / Non-Injured Limb  4 - Symptomatic PE  5 - Combined DVT and PE |
| Location of DVT | <Free Text> |
| Single Event or Multiple | 1 - Single  2 - Multiple |
| Date of VTE | (Date) |
| Time to VTE from Surgery / Achilles Rupture (if non-operative) | (Days) |
| Compliant with VTE Prophylaxis | 0 - No  1 - Yes |
| Post Surgery / Injury (Achilles) bleeding event | 0 - No  1 - Yes |
| Heparin Induced Thrombo-cytopenia | 0 - No  1 - Yes |
| Post Surgery Infection | 0 - None  1 - Superficial  2 - Deep |
| Post Surgery Wound complication | 0 - No  1 - Yes |
| Mortality within 90 days | 0 - No  1 - Yes |
| Date of Mortality | (Date) |
| Co-morbidities  (All to be answered as No / Yes) | Smoker  Asthma / COPD  Active / actively treated Malignancy  Chronic Kidney Disease  Hypertension  Other Cardiac Conditions (MI / NSTEMI / CCF / Angina, etc.)  Dementia  Diabetes  Peripheral Vascular Disease  Clotting Disorder  Previous History of VTE  Recent Long-Distance Travel (previous 2 weeks)  Pregnancy  Stroke |
| Other Co-morbidities | <Free Text> |
| Other Comments / Clarifications | <Free Text> |
| Multiple Operations | (Number)  <Included qualifiers to match patients> |

# **Appendix 2:** Regression Analysis

| **Case Processing Summary** | | | |
| --- | --- | --- | --- |
| Unweighted Cases | | N | Percent |
| Selected Cases | Included in Analysis | 2840 | 85.8 |
|  | Missing Cases | 469 | 14.2 |
|  | Total | 3309 | 100.0 |
| Unselected Cases | | 0 | .0 |
| Total | | 3309 | 100.0 |

| **Categorical Variables Coding** | | Frequency | Parameter coding |
| --- | --- | --- | --- |
| Foot and Ankle Procedures | Elective | 2316 | 0 |
|  | Trauma (excl. Achilles Ruptures) | 445 | 1 |
|  | Acute Diabetic Foot | 73 | 2 |
|  | Achilles Ruptures (undergoing surgery) | 2 | 3 |
|  | Achilles Ruptures (non-operative) | 4 | 4 |
| ASA Grade | Grade I | 940 | 0 |
|  | Grade II | 1590 | 1 |
|  | Grade III - V | 310 | 2 |
| Current Smoker | No | 2592 | 0 |
|  | Yes | 248 | 1 |
| Clotting disease | No | 2826 | 0 |
|  | Yes | 14 | 1 |
| Previous History of VTE | No | 2809 | 0 |
|  | Yes | 31 | 1 |
| Recent Long-Distance Travel | No | 2833 | 0 |
|  | Yes | 7 | 1 |
| Post-operative splintage | No | 2195 | 0 |
|  | Yes | 645 | 1 |
| Operation Type: Open Surgery | No | 324 | 0 |
|  | Yes | 2516 | 1 |
| Post-operative Weight Bearing Status | Not weight bearing | 226 | 0 |
|  | Full or partial weight bearing | 2614 | 1 |
| Post-operative Mechanical Prophylaxis  (Contralateral side) | No | 1556 | 0 |
|  | Yes | 1284 | 1 |
| Torniquet used | No | 561 | 0 |
|  | Yes | 2279 | 1 |
| Surgery Urgency:  Immediate, Urgent or Expedited | No | 2296 | 0 |
|  | Yes | 544 | 1 |

| **Model Summary** | | | |
| --- | --- | --- | --- |
| Step | -2 Log likelihood | Cox & Snell R Square | Nagelkerke R Square |
| 1 | 88.594 | .019 | .391 |
|  | | | |

| **Variables in Final Equation** | Sig. | Exp(B) | 95% C.I.for EXP(B) | |
| --- | --- | --- | --- | --- |
|  |  |  | Lower | Upper |
| Age (Years) | .709 | .990 | .942 | 1.042 |
| ASA Grade | .535 |  |  |  |
| ASA Grade (1) | .264 | 3.719 | .371 | 37.285 |
| ASA Grade (2) | .428 | 3.663 | .147 | 91.151 |
| Number of Co-morbidities | .815 | 1.109 | .464 | 2.652 |
| Current Smoker (1) | .905 | 1.143 | .128 | 10.181 |
| Clotting disease (1) | .999 | .000 | .000 | . |
| Previous History of VTE (1) | .998 | .000 | .000 | . |
| Recent Long-Distance Travel (1) | .999 | .000 | .000 | . |
| Surgery Urgency: Immediate, Urgent or Expedited (1) | .664 | 1.888 | .108 | 33.146 |
| Foot and Ankle Procedures | .959 |  |  |  |
| Foot and Ankle Procedures (1) | .425 | 3.280 | .178 | 60.506 |
| Foot and Ankle Procedures (2) | .997 | .000 | .000 | . |
| Foot and Ankle Procedures (3) | 1.000 | 3.113 | .000 | . |
| Foot and Ankle Procedures (4) | .997 | 1.885 E+25 | .000 | . |
| Operation Type: Open Surgery (1) | .994 | 3.083 E+6 | .000 | . |
| Torniquet used (1) | .964 | 1.043 | .163 | 6.683 |
| Post-operative Mechanical Prophylaxis (contralateral side) (1) | .867 | 1.141 | .246 | 5.297 |
| Post-operative Weight Bearing Status (1) | .995 | 5.281 E+6 | .000 | . |
| Post-operative splintage (1) | .594 | .556 | .064 | 4.807 |
| Constant | .991 | .000 |  |  |
