# Supplementary material for: Characteristics of Patients Not Receiving Chemical Thromboprophylaxis Following Foot and Ankle Surgery: Data From the Multicenter, Prospective UK Foot and Ankle Thrombo-Embolism Audit (UK-FATE)
Source: Foot Ankle Int. 2024 Jun 13;45(9):943–9. doi: 10.1177/10711007241258159 (PMC11408974; doi:10.1177/10711007241258159)
Supplement: sj-docx-4-fai-10.1177_10711007241258159 – Supplemental material for Characteristics of Patients Not Receiving Chemical Thromboprophylaxis Following Foot and Ankle Surgery: Data From the Multicenter, Prospective UK Foot and Ankle Thrombo-Embolism Audit (UK-FATE) [file sj-docx-4-fai-10.1177_10711007241258159.docx]

Characteristics of patients not receiving chemical thromboprophylaxis following foot and ankle surgery – Data from the multicenter, prospective UK Foot and Ankle Thrombo-Embolism Audit (UK-FATE)

**List of Collaborators**

# **Supplementary Data:** Participating Sites and Investigators

| Site | Contributors |
| --- | --- |
| Barking Havering & Redbridge NHS Trust | Rabia Shaikh / Wilam Alfred / Imobhio Okhifun / Ece Cinar / Nelson Bua / Krishna Vemulapalli / Ashok Acharya |
| Barnsley Hospital NHS Foundation Trust | Richard Gadd / John Money-Taylor / Rohit Kantharaju / Abhijit Bhosale / Suchita Bahri / Rosie Broadbent |
| Barts Health - Royal London | Isabella Drummond / Neil Jones / Savan Shah / Thuwarahan Ravindrarjah / Zaid Yasen / Kunjshri Singh / Ruqaiya Al-Habs |
| Barts Health - Whipps Cross | Lucky Jeyaseelan / Abdullah Habbiba / Thomas Walker / Maximilian Dewhurst / Nisha Glasgow / Dominic Eze / Gary Carter |
| Basildon University Hospital | Praveen Rajan / Vijay Patil / Omer Amer / Kalim Malik / Pranavan Pavanerathan / Arijit Mallick / Ilias Seferiadis |
| Bedfordshire Hospitals NHS Foundation Trust - Luton & Dunstable | Verity Currall / Preetha Sadasivan / Sunil Kumar / Shahrukh Reza Sanjani / Maria Ciaccio |
| Blackpool Teaching Hospitals NHS Trust | Brijesh Ayyaswamy / Pradeepsyam Prasad / Mr Anand / Dr Sunilraj / Suzanne Lane / Swetha Prathap |
| Bucks Healthcare | Raghubir Kankate / Ioannis Aktselis / Kinner Davda / Arvind Vijapur / Mohammed Tayyem / Jackie Chau |
| Calderdale and Huddersfield NHS Trust | Muhammad Saad Azhar / Simon Sturdee / Halima Hussain / Sarah Sonde / Muhammad Qais Luqman / Rahy Farooq / Gareth Wells / Aneil Shenolikar |
| Cardiff and Vale UHB | Michiel Simons / Paul Hodgson / Rhys Thomas / Sam Stevens |
| County Durham Darlington Foundation Trust | Yahya Elhassan / Adebowale Adeniyi / Will Aspinall |
| Cwm Taf Morgannwy UHB - Prince Charles Hospital | Vinay Joseph / Miriam Day |
| Cwm Taf Morgannwg UHB - Royal Glamorgan Hospital | Aureola Tong / Claire Joyner |
| Doncaster and Bassetlaw Teaching Hospitals NHS Foundation Trust | Muhammed Alzaranky / Osman Elhassan / Kishor Chhantyal / Abhishek Arora / Zain Abiddin / Robert Kucharski |
| Dudley Group of Hospitals NHS Trust | Irfan Ahmad / Junaid Zeb / Usman Ishaq / Jija Thomas / Kowshik Jain |
| East & North Herts NHS Trust | Rupinderbir Deol / Rad Faroug / Karan Johal / Simon Mordecai / Miltiadis Argyropouos / Amit Chawla / Mohamed Ibrahim / Marta Pereira |
| East Suffold & North Essex Hospitals | Lynne Barr/ Elda Julies / Francesca Hill |
| East Sussex Healthcare NHS Trust | Smriti Kapoor / James Bailey / Ishani Mukhopadhyay / Sarina Rana / Hamza Tarig / Mahdi Qualaghassi / Sheena Seewoonarian / Barry Rose |
| Epsom and St Helier NHS Trust | Georgina Crate / Sarah Abbott / Christopher Fenner / Ryan Geleit / Sohail Yousaf |
| Frimley Health NHS Foundation Trust - Wexham Park Hospital | Nimra Akram / Zahra Al-Hubeshy / Bhavi Patel / Mohamed Hussein / Callum Clark / Jasdeep Giddie / Raman Dega |
| George Eliot NHS Trust | Kishore Dasari / Gurbinder Nandhara / Pritesh Kumar / Prateek Gupta / Hope Poole / Pamela Zace |
| Glan Clwyd Hospital (Betsi Cadwaladr University Health Board) | Farhan Alvi / Jagan Jacob / Raji Reddy / Vaishnav Sateesh |
| Great Western Hospital Trust | Andrea Gledhill / James Craven / Matt Cichero / Ben Yates / Ayla Newton / John Grice |
| Hampshire Hospitals NHS Foundation Trust | Nicholas Fawcett / Hossam Fraig / Farouk Hamad / Daniel Marsland / Robin Elliot |
| Homerton Healthcare NHS Foundation Trust | Yaser Ghani / Suresh Chandrashekhar / Ravi Kant Millan |
| Imperial College Healthcare Trust - St Mary's | Andrew Clark / Kashed Rahman / Mark Sykes |
| Kingston Hospital | Zoe Little / Jawaad Saleem / Lewis Jolly / Aman Jain / Ansar Qadri |
| Lancashire Teaching Hospitals NHS Trust | Sophy Rymaruk |
| Lincoln County Hospital, United Lincolnshire NHS Trust | Avadhut Kulkarni / Mohanrao Garabadi / Meraj Akhtar / Munier Hossain / Shamael Yunus / Maleeha Saleem / Joanna Fong |
| Liverpool University Hospitals NHS Foundation Trust | Amirul Islam / Ben Nusir / James Chapman / David Holmes / Neville Mamoowala / Kieran Almond / Claire Wright |
| Manchester University Hospitals - MRI & Trafford | Ethan Caruana / Thomas Watson / Georgia Allison |
| Manchester University Hospitals - Wythenshawe Hospital | Anand Pillai / Imad Madhi / Mazin Alsalihy / Khadija Elamin / Chee Rong Yip / Lucy Tew / Rohan Dahiya |
| Mid Yorkshire NHS Trust | Thomas Goff / Oliver Bagshaw / Henry Slade / Paul Andrzejowski / Ayoub Gomati / Chris Drake |
| Milton Keynes University Hospital | Jamie Hind / Rebecca Morgan / Ahmed Khalaf / Adeel Ditta / Arul Ramasamy |
| NHS Fife | Joshua McIntyre / Calum Blacklock / Scott Middleton / Robert Clayton |
| NHS Greater Glasgow & Clyde | Alex Hrycaiczuk / Christopher Thornhill / Gowsikan Jeyakumar / Delani Vaithilingam / Kate Potter / Bilal Jamal /Pete Chan |
| NHS Highland / Raigmore Hospital | Muyed Mohamed / Debbie Fraser / Ahmed Elhalawany / James Beastall / Gerard Cousins / Perrico Nunag |
| Norfolk and Norwich University Hospitals | David Loveday / Akshdeep Bawa / Rebecca Gilmore / Kerstin Schankat |
| North Bristol NHS Trust | Andrew Walls / Nicole Corin / Peter Robinson / Steve Hepple / William Harries / Andrew Riddick / Ian Winson / Luke Marsh |
| North Middlesex University Hospital | Muhammad Amer Bashir / Jigyasa Saini / Henry Atkinson |
| North Tees and Hartlepool NHS Foundation Trust | Rajiv Limaye / Sarah Johnson-Lynn / Mohit Sethi |
| Northamptonshire Healthcare NHSFT | George Flanagan / Akram Uddin / Ian Reilly |
| Northumbria Healthcare NHS Trust | Rebecca Martin / Andrea Pujol-Nichol / Natalie Carroll |
| Nottingham University Hospital | Alexander Boucher / Mustafa Alward / Yuland Myint |
| Oxford University Hospitals NHS Trust - Nuffield Orthopaedic Centre | Katherine Butler / Adrian Kendal / Mark Bugeja / Justin Mooteeram / Farid Saedi |
| Portsmouth Hospitals University NHS Trust - Queen Alexandra Hospital | Togay Koc / Zeid Morcos / Gregory Robertson / Natal Holmes / Howard Tribe / Tim Pearkes / Ahmed Soliman |
| Practice Plus Group, Ilford | Anil Prasanna |
| Princess Alexandra Hospital NHS trust | Kar Teoh / Sanil Kamat / Abhijit Bajracharya / James Reeves / Mbori Ngwayi / Galal Imtiaz / Noah Blackmore |
| Princess Royal University Hospital / King's NHS Trust | Benjamin Lau / Arjun Naik / Eleanor Tung / Siddhartha Murhekar / Robbie Ray / Shirley Lyle |
| Robert Jones and Agnes Hunt Hospital | Nilesh Makwana / Kahlan Al Kaisi / Musab Al-Musabi |
| Royal Devon and Exeter | Mike Dean / Adrian Hughes / Kimberley Shuttlewood |
| Royal National Orthopaedic Hospital | Matthew Welck / Shelain Patel / Adam Sykes / Mahesh Mamballi Thibbaiah / Hosain Hadi / Anil Haldar / Amir Gahanbani Ardakani / Priyanka jani |
| Royal Oldham Hospital, Northern Care Alliance | Vladislav Kutuzov / James Gibbons / Daniel Trussler / Eve Hawley / Sabeen Akhtar |
| Royal Orthopaedic Hospital NHS Trust | Harshadkumar Dhirajlal Rajgor / Basil Budair / Hari Prem / James Mckenzie / Daniel Thurston / Michael O'Sullivan / Mohammed Elmajee / Erika Pond |
| Royal Shrewsbury Hospital | Wajiha Zahra / Catriona Heaver |
| Royal Surrey Hospital, Guildford | Kueni Igbagiri / Andrew Gaukroger / Matthew Solan / Christian Peacock / Ka Siu fan |
| Royal United Hospital Bath NHS Foundation Trust | Tristan Barton / Derek Robinson / Selina Graham / Julian Zeolla |
| Somerset NHS Foundation Trust - Taunton Site | Samuel Everett / Mohammad Iqbal / Lysander Gourbault / Shashwat Singh / Cary Tang / Mariam Tarhini |
| South Tyneside and Sunderland NHS Foundation Trust | Shahrukh Khan / Satishkumar Balasubramanian |
| Southport and Ormskirk Hospital NHS Trust | Caroline Lever / Vaibhav Bansod / Kartik Iyengar / Abdul Wadood / Lara McMillan / Eugene Toh |
| St Richards Hospital, Chichester | Stanley Masunda / Simon Federer / Faheem Ahmad / Ahmed Lashin / Ahmed Kaddah / Emmanuel Oladeji / Ed Dawe |
| University Hospital Coventry and Warwickshire | Ciaran Nolan / Khalil El-Bayouk / Vivek Dhukaram / Anna Chapman |
| University Hospital Dorset (Bournemouth and Poole) | Laura Beddard / Alex Thomas / Vipul Garg / Heath Taylor / Nikki Kelsall / Charline Roslee |
| University Hospitals of Leicester | Nimra Akram / Hamish Lowdon / Sherif Ahmed-Kamel / Anette Jones / Alistair Best / Mate Zabaglo |
| University Hospitals Sussex NHS Trust Worthing | Junaid Sayani / Olive Kyaw / Chan Khin / Ramla Ali / Yousufuddin Shaik / Natasha Hossain / Lucia Valente / Adam Ajis |
| Worcestershire Acute Hospitals NHS Trust | Abhijit Guha / Melwyn Pereira / Atif Ayoub / Vlad Paraoan / Nayeem Hali / Charles Baird / Raj Kugan / Ahmad Abdallatif |
| Wirral University Teaching Hospitals | Mark Blomfield / Gillian Jackson / James Craven / Anubhav Malhotra / Aileen Toner / Luke Render / Connor Ashley / Richard Limb |
| Wrightington Wigan and Leigh NHS Foundation Trust | Robert Smith / Luke Hughes |
| York and Scarborough Hospitals NHS Trust | Hannah Matthews / Fleur Shiers-Gelalis / Jason Ting / Stuart Place / Adam Budgen / James Stanley / Charlie Jowett |
